# A Computational Module Assembled from Different Protease Family Motifs Identifies PI PLC from *Bacillus cereus* as a Putative Prolyl Peptidase with a Serine Protease Scaffold

**DOI:** 10.1371/journal.pone.0070923

**Published:** 2013-08-05

**Authors:** Adela Rendón-Ramírez, Manish Shukla, Masataka Oda, Sandeep Chakraborty, Renu Minda, Abhaya M. Dandekar, Bjarni Ásgeirsson, Félix M. Goñi, Basuthkar J. Rao

**Affiliations:** 1 Unidad de Biofísica (Consejo Superior de Investigaciones Científicas, Universidad del Pais Vasco/Euskal Herriko Unibertsitatea) and Departamento de Bioquímica, Universidad del País Vasco, Bilbao, Spain; 2 Department of Biological Sciences, Tata Institute of Fundamental Research, Homi Bhabha Road, Mumbai, India; 3 Department of Microbiology, Faculty of Pharmaceutical Science, Tokushima Bunri University, Tokushima, Japan; 4 Department of Biological Sciences, Tata Institute of Fundamental Research, Mumbai, India; 5 Department of Biological Sciences, Tata Institute of Fundamental Research, Mumbai, India; 6 Plant Sciences Department, University of California, Davis, Davis, California, United States of America; 7 Science Institute, Department of Biochemistry, University of Iceland, Dunhaga, Reykjavik, Iceland; 8 Unidad de Biofísica (Consejo Superior de Investigaciones Científicas, Universidad del Pais Vasco/Euskal Herriko Unibertsitatea) and Departamento de Bioquímica, Universidad del País Vasco, Bilbao, Spain; 9 Department of Biological Sciences, Tata Institute of Fundamental Research, Mumbai, India; University of South Florida College of Medicine, United States of America

## Abstract

Proteolytic enzymes have evolved several mechanisms to cleave peptide bonds. These distinct types have been systematically categorized in the MEROPS database. While a BLAST search on these proteases identifies homologous proteins, sequence alignment methods often fail to identify relationships arising from convergent evolution, exon shuffling, and modular reuse of catalytic units. We have previously established a computational method to detect functions in proteins based on the spatial and electrostatic properties of the catalytic residues (CLASP). CLASP identified a promiscuous serine protease scaffold in alkaline phosphatases (AP) and a scaffold recognizing a β-lactam (imipenem) in a cold-active *Vibrio* AP. Subsequently, we defined a methodology to quantify promiscuous activities in a wide range of proteins. Here, we assemble a module which encapsulates the multifarious motifs used by protease families listed in the MEROPS database. Since APs and proteases are an integral component of outer membrane vesicles (OMV), we sought to query other OMV proteins, like phospholipase C (PLC), using this search module. Our analysis indicated that phosphoinositide-specific PLC from *Bacillus cereus* is a serine protease. This was validated by protease assays, mass spectrometry and by inhibition of the native phospholipase activity of PI-PLC by the well-known serine protease inhibitor AEBSF (IC50 = 0.018 mM). Edman degradation analysis linked the specificity of the protease activity to a proline in the amino terminal, suggesting that the PI-PLC is a prolyl peptidase. Thus, we propose a computational method of extending protein families based on the spatial and electrostatic congruence of active site residues.

## Introduction

Proteolytic enzymes catalyze the cleavage of peptide bonds in proteins and are divided into several major classes based on their mechanism of catalysis [1], [2]. The MEROPS database systematically categorizes these protein families and clans to provide an integrated information source [3]. The abundance of proteolytic enzymes in biological systems results from the varied physiological conditions under which these enzymes have evolved to be effective [4].

We selected proteases with known active sites and 3D structures from each family listed in MEROPS and encapsulated their active site motifs into a single protease search module. We previously presented a bottom-up method for active site prediction (CLASP) using active site residues [5]. Subsequently, we used CLASP to quantify promiscuous activities in a wide range of proteins [6]. Here, we used CLASP to query proteins of interest for proteolytic function using this search module. Such a search module is equivalent to running a BLAST search from the MEROPS database site [7], [8].

While BLAST looks for sequence homology, CLASP detects spatial and electrostatic congruence between residues to predict similar catalytic properties in proteins. Sequence alignment techniques are known to fail to detect distant relationships since considerable divergence often resembles noise [8]. More importantly, proteins redesigned from chiseled scaffolds through exon shuffling and those resulting from convergent evolution remain beyond the scope of such methods [9]. The phenomenon of convergent evolution, first proposed in serine proteases [10], is no longer considered to be a rare event [11], [12]. Structural alignment methods have addressed some of these deficiencies, but can be misled by non-catalytic parts of the protein [13]. A recent method employs learning techniques to predict whether proteins have proteolytic activities, but has not identified any novel proteases undetected by other methods [14], [15]. CLASP unraveled a promiscuous serine protease scaffold in alkaline phosphatases (AP) [5], one of the widely studied promiscuous enzyme families [16], [17], and also a scaffold recognizing a β-lactam (imipenem) in a cold-active *Vibrio* AP [18], [19].

Several conserved proteases have been implicated in bacterial pathogenesis [20]. Proteases are integral components of outer membrane vesicles (OMVs), which all gram-negative bacteria shed as blebs from the cell surface [21]. We queried other proteins present in OMVs using the CLASP protease search module and found that phosphoinositide-specific phospholipase C (PI-PLC) is a Pro-X specific protease. PI-PLCs are part of the signal transduction pathways of higher organisms [22]–[24]. Prokaryotic PI-PLCs are important virulence factors that alter the signaling pathways of higher organisms [25]–[27]. We demonstrated a serine protease domain in PI-PLC from *Bacillus cereus* through its proteolytic activity and the inhibition of its native activity on phospholipids by serine protease inhibitors (IC50 = 0.018 mM). Edman degradation analysis demonstrated that the specificity of the protease activity was for a proline in the amino terminal, suggesting that PI-PLC is a prolyl peptidase [28].

To summarize, the distinct types of proteases categorized in the MEROPS database were used to generate a search module that could be used to query any protein with known 3D structure for the presence of a promiscuous proteolytic activity. This search module identified a serine protease scaffold in PI-PLC from *Bacillus cereus*, which was validated by *in vitro* experiments. A similar computational approach can be adopted for other enzymatic functions to extend protein families based on the spatial and electrostatic congruence of active site residues: relationships that often escape detection by sequence alignment or global structure alignment methods.

## Results

We chose a set of proteases with known 3D structures and active site residues from each of the seven major classes in the MEROPS database (Table 1) [3]. We then created signatures encompassing the spatial and electrostatic properties of the catalytic residues in these proteins [5]. To maintain uniformity, we chose three residues from the active site neighborhood, including the catalytic residues (Table 2). These signatures were then used to query other proteins of interest using CLASP. Matches with low scores (less than an empirical threshold of 0.1) indicate a good spatial and electrostatic congruence, and a significant likelihood that these proteins possess proteolytic functions.

**Table 1:** Proteases from different families.

PDB	Sequencelength	Function	Type
1FLH	326	Uropepsin	A
2CY7	396	Cysteine protease APG4B	C
1S2B	206	Eqolisin family of peptidases	G
1FJO	316	Thermolysin	M
1VDE	454	Homing endonuclease	N
1A0J	223	Trypsin	S
2DBU	366	Gamma-glutamyltranspeptidase	T

Motifs extracted from each of these proteases consist of three residues. Types: aspartic (A), cysteine (C), glutamic (G), metallo (M), asparagine (N), serine (S), threonine (T).

**Table 2:** Active site residues, distances (D), and potential difference (PD) of residue pairs for proteins from each major class in the MEROPS database.

PDB	Motif			D (Å)			PD		
	a	b	c	ab	ac	bc	ab	ac	bc
1FLH	ASP32	ASP215	GLY34	2.933	2.779	3.461	−30	−293	−262
2CY7	CYS74	ASP278	HIS280	7.723	3.413	4.73	331	185	−146
1S2B	GLN53	GLU136	TRP39	7.013	6.026	5.059	130	−45	−176
1FJO	HIS142	GLU143	HIS146	4.868	3.162	4.122	−61	30	92
1VDE	ASN454	CYS1	HIS79	6.028	6.983	5.156	−182	−171	11
1A0J	ASP102	SER195	HIS57	7.844	5.567	3.314	−144	−39	104
2DBU	THR391	ASN411	TYR444	6.797	6.219	2.613	389	−39	−429

Potential differences are in units of kT/e (k is Boltzmann's constant, T is the temperature in K and e is the charge of an electron).

To expand our previous work on APs, we investigated the proteolytic activity of a cold-active *Vibrio* AP (VAP) [18] on four substrates: benzoyl-Arg-pNA, Z-GlyProArg-pNA, succinyl-AlaAlaAla-pNA, and succinyl-AlaAlaProPhe-pNA. While we detected no proteolytic activity in VAP, its native AP activity was inhibited by AEBSF (4-(2-aminoethyl) benzenesulfonyl fluoride hydrochloride) (IC_50_ of 0.35+/−0.05 mM (n = 6) for AEBSF at pH 7.0), but not by PMSF (phenylmethanesulfonylfluoride or phenylmethylsulfonyl fluoride). Both AEBSF and PMSF are serine protease inhibitors with similar specificity (chymotrypsin, kallikrein, plasmin, thrombin, and trypsin).

**Figure 1:**
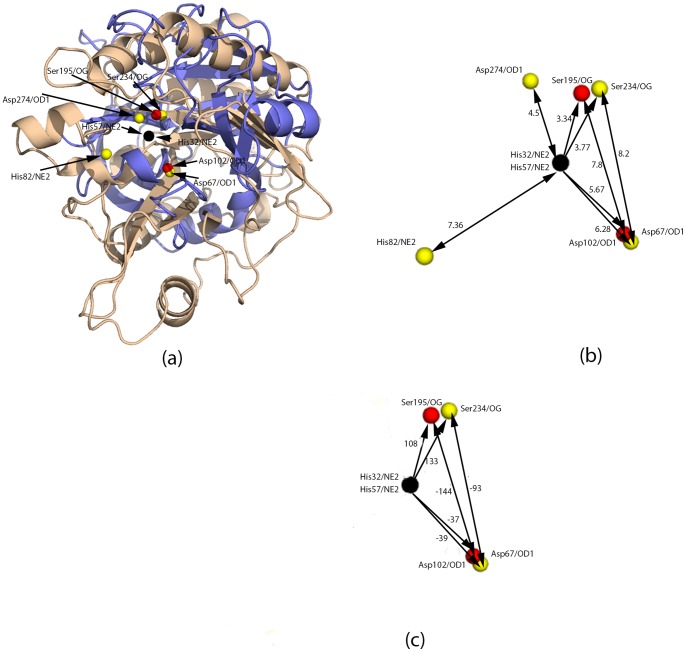
Superimposed active sites of trypsin and PI-PLC based on the active site match: His/57/NE2, Asp/102/OD1, and Ser/195/OG from PDBid:1A0J and His/32/NE2, Asp/67/OD1, and Ser/234/OG from PDBid:1PTD, respectively. (a) Superimposed proteins. Trypsin (PDBid:1A0J) is in blue and PI-PLC (PDBid:1PTD) is in grey. After superimposition, all three atoms in both proteins lie on the same plane (Z = 0), such that His57 and His32 (colored in black) lie on the coordinate center and Asp102 and Asp67 lie on the X-Y plane (Y = 0). The active site residues of trypsin are red and those of PI-PLC, yellow. His32, Asp67, His82, and Asp274 are all part of the active site scaffold in PI-PLC [30]. (b) Distances between pairs of residues in the matches in Å. (c) Potential differences between pairs of residues in the matches. Electrostatic potential in dimensionless units of kT/e where k is Boltzmann’s constant, T is the temperature in K and e is the charge of an electron.

The predicted residues, deviations in distances, potential difference in cognate pairs, and scores were determined for a phosphoinositide-specific PLC (PI-PLC) (PDB id: 1PTD) from *Bacillus cereus* (Table 3). PI-PLC was indicated to be a serine protease because the best match was with a trypsin protein, PDBid:1A0J [29]. The residues predicted by CLASP as responsible for its protease activity coincide with the active site responsible for its native phospholipase activity (His32, Asp67, His82, and Asp274) (Fig. 1) [30]. However, there was little sequence similarity within the set of querying and queried proteins, suggesting that established sequence alignment methods would fail to detect this relationship (Table S1).

**Table 3:** The deviation in distances (δD), potential difference in cognate pairs (δPD), predicted residues (PR), and final scores of a PI-PLC (PDB id: 1PTD) from *Bacillus cereus*.

PDB	PR			δD (Å)			δPD			Scores
	a	b	c	ab	ac	bc	ab	ac	bc	
1FLH	ASP153	ASP19	GLY152	−6.4	−2.2	−2.1	−46.1	−62.4	−16.4	55
2CY7	–	–	–	–	–	–	–	–	–	–
1S2B	GLN286	GLU287	TRP10	−0.2	1.3	−4.9	−41.5	43.5	85.1	24
1FJO	HIS32	GLU117	HIS82	−3.9	−4.2	−3	47.7	−79.3	−127	71
1VDE	–	–	–	–	–	–	–	–	–	–
1A0J	ASP67	SER234	HIS32	−0.3	−0.6	−0.4	−50.4	−78.9	−28.6	0.07
2DBU	THR218N	ASN221	TYR229	0.1	1.2	0	122.5	−180.6	−303.1	303

**Figure 2:**
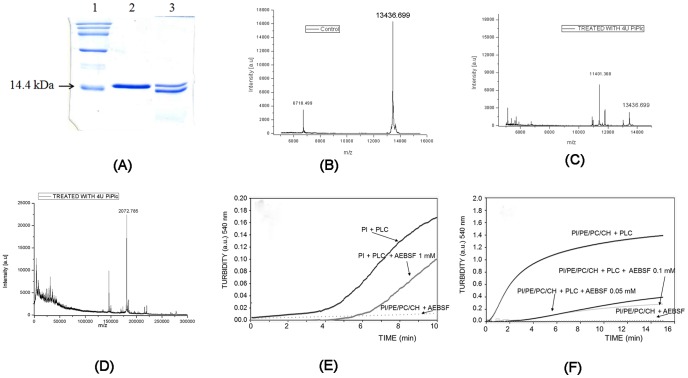
Confirming the protease scaffold in PI-PLC by proteolytic assays and inhibition studies. (A) Protease activity of PI-PLC. Substrate protein (UVI31+, lane 2) was incubated with PI- PLC (lane 3) overnight at 37°C, followed by sample analysis with 15% SDS-PAGE. Lane 1, molecular weight marker. (B) Control for UVI31+, with peak at 13.436 kDa. (C) UVI31+ treated with PI-PLC, showing fragmented peaks at 11.4 kDa and (D) another fragment of 2.0 kDa. **(**E**)** The inhibition of PI-PLC activity on phosphatidylinositol (PI) by trypsin inhibitor AEBSF. (F) The inhibition of PI-PLC activity on PI by trypsin inhibitor AEBSF in a mixture with phosphatidylcholine (PC), phosphatidylethanolamine (PE), and cholesterol (CH).

We tested this prediction by performing an *in vitro* protease assay on commercially available PI-PLC from *Bacillus cereus*. The protease activity of PI-PLC on the substrate protein UVI31+ [31], [32] was inhibited by the protease inhibitor leupeptin, while other inhibitors like AEBSF were unstable during a long incubation (Fig. 2A). A MALDI TOF analysis showed a clean, 13.4 kDa peak for purified UVI31+ protein (Fig. 2B), which was split into two fragments of 2.0 kDa (Fig. 2C) and 11.4 kDa (Fig. 2D) on incubation with PI-PLC. Edman degradation analysis demonstrated that the protease activity was specific for a proline following the first seven residues of the UVI31+ protein (marked by an asterisk - MAEHQLGP*IAG). This suggested that the PI-PLC is a putative prolyl peptidase. The predicted protease scaffold was tested by assaying inhibition of its phospholipase activity by the trypsin inhibitor AEBSF (IC_50_ = 0.018 mM). Assays were performed with the substrate in the form of large, unilamellar vesicles. The vesicles consisted of either pure phosphatidylinositol (PI) (Fig. 2E) or an equimolar mixture of PI, phosphatidylcholine (PC), phosphatidylethanolamine (PE), and cholesterol (CH) (Fig. 2F). In both cases, the maximum reaction rates decreased in a dose-dependent way in the presence of AEBSF (Fig. S1).

We tested the proteolytic functions and inhibition using protease inhibitors of the non-toxic *Bacillus cereus* phosphatidylcholine-specific phospholipase C (PC-PLC) and the closely related highly toxic *C. perfringens* α-toxin (CPA), which possesses an additional C-terminal domain responsible for the sphingomyelinase, hemolytic, and lethal activities [33]. CPA and PC-PLC activity on phospholipids was unaffected by trypsin inhibitors, consistent with the CLASP analysis which fails to detect a serine protease scaffold in these proteins (Table 4, 5).

**Table 4:** The deviation in distances (δD), potential difference in cognate pairs (δPD), predicted residues (PR), and final scores for *C. perfringens* α toxin (CPA) (PDB id: 1CA1).

PDB	PR			δD (Å)			δPD			Scores
	a	b	c	ab	ac	bc	ab	ac	bc	
1FLH	ASP298	ASP293	GLY296	−0.7	−2.4	−2.6	−89.6	−49.8	39.8	41
2CY7	CYS169	ASP25	HIS241	−0.5	−11.9	−8.6	11.5	−33.7	−45.2	135
1S2B	GLN110	GLU108	TRP109	−2.4	−3.7	−5.6	67	104.4	37.5	52
1FJO	HIS136	GLU152	HIS148	−0.1	−0.6	0.2	10.7	87	76.2	0.08
1VDE	ASN172	CYS169	HIS241	−1.8	−3.5	−10.1	45.1	−162.8	−207.9	262
1A0J	ASP216	SER209	HIS212	−1.7	−1.6	0	68.8	−61	−129.9	13
2DBU	THR272N	ASN297	TYR307	−0.1	−3.5	−6.8	156.1	−104	−260.3	339

**Table 5:** The deviation in distances (δD), potential difference in cognate pairs (δPD), predicted residues (PR), and final scores for a PC-PLC (PDB id: 1AH7) from *Bacillus cereus*.

PDB	PR			δD (Å)			δPD			Scores
	a	b	c	ab	ac	bc	ab	ac	bc	
1FLH	ASP72	ASP74	GLY76	−2	−2.5	−3.8	−126.5	−25.3	101.2	28
2CY7	–	–	–	–	–	–	–	–	–	–
1S2B	GLN39	GLU42	TRP43	0.6	0.1	1.3	−13.3	−64.4	−51	0.1
1FJO	HIS118	GLU146	HIS14	−3	−0.2	−1.9	−128.2	−49.6	78.5	15
1VDE	–	–	–	–	–	–	–	–	–	–
1A0J	ASP55	SER2	HIS14	−1.5	0.3	−3.5	49.8	21.3	−28.6	26
2DBU	THR151N	ASN155	TYR156	−3.1	−1.5	−3	274.5	−53.6	−328.2	369

CPA does have a metallo-protease motif from thermolysin PDBid:1FJO (Table 4). Remnants of a metallo-protease in the CPA protein preparation prevented direct confirmation of its proteolytic function. A metallo-protease inhibitor did not inhibit CPA activity. This lack of inhibition by a single compound is insufficient to rule out the existence of a metallo-protease scaffold.

**Figure 3:**
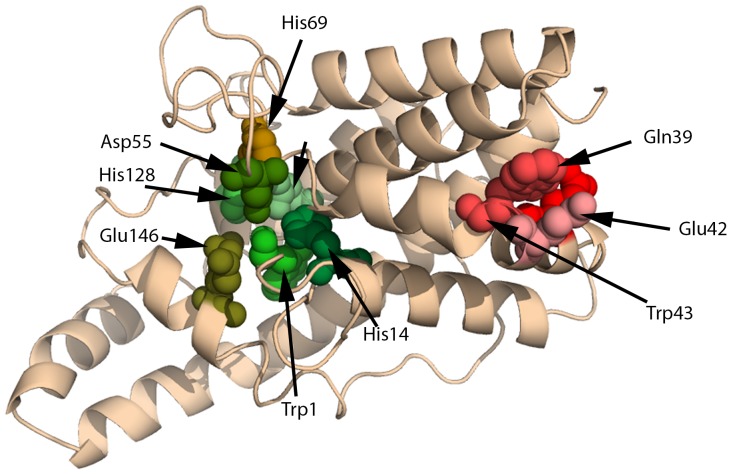
CLASP detects a glutamic protease motif in PC-PLC (PDBid:1AH7). The residues predicted to be responsible for the protease activity (Gln39, Glu42, and Trp43, in shades of red) does not coincide with its native active site (Trp1, His14, Asp122, HiS128, Glu146, Asp55, and His69, in shades of green). The motif is selected from a protein from the Eqolisin family of peptidases: PDBid:1S2B.

The PC-PLC proteolytic activity could also be an artifact of metallo-protease contamination, which is difficult to remove. CLASP detects in this protein a glutamic protease motif from the Eqolisin family of peptidases, PDBid:1S2B (Table 5), which does not coincide with its native active site (Fig. 3). While this protein’s lack of inhibition by serine and metallo-protease inhibitors is consistent with CLASP analysis, mutational studies would be required to confirm the moonlighting glutamic protease scaffold [34]. Thus, the protease activities of CPA and PC-PLC remain open to debate.

## Discussion

Proteases have evolved to use different mechanisms for proteolysis [2], [3], [35]–[37]. Although most peptidases cleave peptide bonds by hydrolysis, recently a novel protease was shown to be a lyase [38], [39]. There is considerable interest in developing computational methods to identify new proteolytic enzymes and their substrates. MEROPS provides a BLAST search for any query protein [3]. Another recent method employed learning techniques to predict proteolytic activities, but found no novel proteases undetected by other methods [14], [15]. Computational methods are also used for predicting protease substrates [40]. Here, we selected proteases with known active sites and structures from each family listed in MEROPS, and encapsulated their active site motifs into a single protease search module. Using our previously described method [5], we exploited this search module to unravel proteolytic activities in phosphoinositide-specific PLC (PI-PLC) [23], [24].

The importance of proteases in organisms from all kingdoms is well established. In humans, abnormal proteolysis is linked to pathologies like cancer, stroke, heart attack, and parasite infection [41]–[43]. The complete set of known proteases present in human, chimpanzee, mouse, and rat have been incorporated into the Degradome database [44]. In plants, papain-like cysteine proteases are critical enhancers of immunity [45]. The bactericidal properties of human neutrophil elastase, a serine protease, have been exploited to design a therapeutic chimeric antimicrobial protein that targets the outer-membrane of bacteria and bolsters the innate immune defense system of grapevines against the Pierce’s disease-causing Gram-negative *Xylella fastidiosa* [46]. Several conserved proteases have been implicated in bacterial pathogenesis and are intricately involved in the Type III secretion system [47], quorum sensing [48], motility [49], chaperones for OMV proteins [50], and the protein quality control mechanism essential for degrading unfolded proteins [51].

Proteases are also an integral component of outer membrane vesicles (OMVs), which are shed by all Gram-negative bacteria as blebs from the cell surface [21]. OMVs from pathogenic bacteria are transported through the host plasma membrane by endocytosis [52], [53], and deliver several virulence factors that modulate the host immune system, alter host cell signaling pathways, and aid the colonization of host tissues [54], [55]. OMVs contain other proteins like alkaline phosphatase (AP), phospholipase C (PLC), and β-lactamases [56].

Previously, we detected a promiscuous serine protease scaffold in APs using CLASP [5], and a scaffold recognizing a β-lactam (imipenem) in a cold-active *Vibrio* AP [18], [19]. The theoretical foundation of CLASP is that the electrostatic potential difference (EPD) in cognate pairs of active site residues is conserved in proteins with the same functionality. The significance of EPD was extended to a method for enumerating possible pathways for proton abstraction in the active site [57], compute electrostatic perturbations induced by ligand binding [58], and propose a rational design-flow for directed evolution [59], [60]. Recently, we proposed a methodology for the multiple sequence alignment of related proteins with known structures using electrostatic properties as an additional discriminator and identified mutations that might be the source of functional divergence in a protein family. The active site and its close surroundings contained enough information to infer the correct phylogeny for related proteins [61]. Here, we confirmed the presence of this proteolytic scaffold in a cold-active *Vibrio* AP (VAP) (IC_50_ of 0.35+/−0.05 mM (n = 6) for AEBSF at pH 7.0). Since APs are present in OMVs, we queried other proteins present in OMVs using motifs from different proteases listed in MEROPS. CLASP analysis using the search module (Table 1 and 2) indicated that PI-PLC is a protease with Pro-X specificity (Table 3). This was validated by protease assays, mass spectrometry and by inhibition of the native phospholipase activity by the serine protease inhibitor AEBSF (IC_50_ = 0.018 mM). Edman degradation analysis demonstrated that the protease activity was specific for a proline in the amino terminal, suggesting that the PI-PLC is a prolyl peptidase [28]. Other endogenous proteolytic substrates of PI-PLC might be discovered by liquid chromatography–mass spectrometry-based peptidomics [62].

Enzymes that cleave phospholipids are defined by the site of cleavage as PLA (releasing the fatty acids) or PLC/PLD (releasing the polar head group) [28], [63]. In higher eukaryotes, phosphoinositide-specific PLC (PI-PLC) produces critical secondary messengers for signal transduction pathways [22], [23]. Prokaryotic PI-PLCs are important virulence factors, possibly by altering this signaling pathway [25], [26]. We experimentally demonstrated the serine protease scaffold in PI-PLC from *Bacillus cereus* (Fig. 2). The hypothesis concerned the origin of the diverse peptidase families and the evolutionary pressures that molded each may be reinforced by these new families of proteolytic enzymes [64].

The genus *Clostridium* consists of spore-forming, rod-shaped, Gram-positive bacteria, of which *Clostridium perfringens* is one of the most pathogenic, with hemolytic, dermonecrotic, vascular permeabilization, and platelet-aggregating properties [65]. *C. perfringens* strains are classified into five toxinotypes based on four typing toxins [66]. The *C. perfringens* α toxin (CPA), present in all five toxinotypes, is a zinc-dependent enzyme with both phospholipase C (PLC) and sphingomyelinase (SMase) activity [67]. The N-terminal domain (∼250 residues) is similar to the *Bacillus cereus* phosphatidylcholine-specific phospholipase C (PC-PLC) [33], [68]. The C-terminal domain has an eight-stranded anti parallel β-sandwich motif similar to eukaryotic calcium-binding C2 domains and confers toxicity on the enzyme [69], [70]. The observed protease activities of CPA and PC-PLC remain unconfirmed due to suspected metallo-protease contamination. However, CPA and PC-PLC activity on phospholipids were unaffected in the presence of trypsin inhibitors, corroborating the CLASP analysis failure to detect a serine protease scaffold in these proteins.

Another aspect of catalysis that should be modeled is the flexibility and diversity in the active site scaffold of related enzymes. For example, there are many unconventional serine proteases [36]. The group of residues that can match a particular residue from the input motif can be varied in CLASP, allowing it to model unconventional motifs. While stereochemical equivalence can be hardwired for amino acids with similar properties, there are instances where residues with different properties occupy the same sequence and spatial location and perform the same function. A well-known example is the equivalence of Ser130 and Tyr150 in Class A and C β-lactamases, respectively [71].

The lack of PI-PLC proteolytic activity on the many tested synthetic substrates, and its specificity for UVI31+ protein, indicates that one should exert caution before ruling out protease activity in an enzyme. This is particularly true when a serine protease inhibitor inhibits the native activity, confirming a serine protease-like scaffold (with the classical catalytic triad) in the active site. Serine protease inhibitors are not active on other serine-centric enzymes like serine β-lactamases, or on metallo-enzymes like CPA and PC-PLC. This establishes their specificity for the serine protease scaffold. Proteases are a unique class of enzymes with many possible substrates due to the theoretically infinite number of DNA sequences that could encode proteins with correspondingly infinite folds. Fluorogenic substrate microarrays determine protease substrate specificity using a wide range of fluorogenic protease substrates [72], [73]. Directed evolution strategies can modify the specificities [59], [74], [75]. The “poor specificity conversion” to convert chymotrypsin to trypsin is an example of the difficulty of such an endeavor [76].

We propose a computational methodology to extend protein families based on the spatial and electrostatic properties of the catalytic residues in proteases. The distinct of protease types categorized in the MEROPS database were selected to generate a search module that can query any protein with known structure for the presence of a promiscuous proteolytic activity.

## Methods

### 1 CLASP Algorithm

The CLASP algorithm was described previously [5]. Given the active site residues from a protein with known structure, a signature encapsulating the spatial and electrostatic properties of the catalytic site is used to search for congruent matches in a query protein, generating a score which reflects the likelihood that the activity in the reference protein exists in the query protein. Adaptive Poisson-Boltzmann Solver [77] (APBS) and the PDB2PQR package [78] were used to calculate the potential difference between the reactive atoms of the corresponding proteins. The APBS parameters are set as follows: solute dielectric, 2; solvent dielectric, 78; solvent probe radius, 1.4 Å; temperature, 298 K; and ionic strength, 0. APBS writes out the electrostatic potential in dimensionless units of kT/e where k is Boltzmann’s constant, T is the temperature in K and e is the charge of an electron. All protein structures were rendered by PyMol (http://www.pymol.org/).

### 2 Protein, Substrate, and Reagents

PI-PLC was purchased from Sigma. Trypsin inhibitor from chicken egg white and PMSF (phenylmethylsulfonyl fluoride) were obtained from Roche.

### 3 Protease Assay

Each reaction mixture (30 µL total volume) contained 13 µM purified UVI31+ protein [31], [32] (14 kDa) and 0.2 units PI-PLC in 50 mM ammonium bicarbonate, and was incubated overnight at 37°C. The protein was then denatured by the addition of 7 µL SDS-denaturing solution (200 mM Tris-HCl pH 6.8, 8% SDS (w/v), 40% glycerol (v/v), 4% 2-mercaptoethanol (w/v), 50 mM EDTA pH 8.0, and 0.08% bromophenol blue (w/v) and heating at 100°C for 3 min. The sample was subjected to 15% SDS-PAGE (w/v) followed by staining with Coomassie brilliant blue. To inhibit protease activity of SAP, three different conditions were employed: (i) 0.1% SDS followed by heating at 100°C for 5 min, (ii) 1 mM PMSF, and (iii) 500 ng/mL trypsin inhibitor, before substrate addition. UVI31+ protein (13 µM) was then added as the substrate and residual enzyme activity was measured.

### 4 PI-PLC Assay and Inhibition Using Trypsin Inhibitors

#### 4.1 Vesicle preparation and characterization

The appropriate lipids - Lipids (Phosphatidylinositol/Phosphatidylethanolamine/Phophatidylcholine/Cholesterol - 40∶30∶15∶15 ratio) were mixed in organic solution and the solvent (mixture of chloroform/methanol/hydrochloric acid mixture 200/100/1, by volume) was evaporated to dryness under N_2_. Solvent traces were removed by evacuating the lipids for at least 2 hr. The lipids were then rehydrated in 10 mM Hepes buffer with 150 mM NaCl, pH 7.5. Large unilamellar vesicles (LUV) were prepared from the swollen lipids by extrusion and sized using 0.1 µm Nuclepore filters, as described by Ahyayauch et al. [79]. The average size of LUV was measured by quasi-elastic light scattering using a Malvern Zeta-sizer. Lipid concentration, determined by phosphate analysis, was 0.3 mM in all experiments.

#### 4.2 Aggregation assay

All assays were carried out at 39°C with continuous stirring in 10 mM Hepes buffer (pH 7.5) with 150 mM NaCl and 0.1% BSA for optimum catalytic activity. The enzyme concentration was 0.16 U/mL. Lipid aggregation was monitored in a Cary Varian UV-vesicle spectrometer as an increase in turbidity (absorbance at 450 nm), as described by Villar et al. [80].

### 5 MALDI-TOF Analysis and Edman Degradation

MALDI-TOF mass spectrometric analysis was performed using an UltraFlextreme MALDI-TOF (Bruker Daltonics, Germany). Positive ionization and linear mode were used. The experimental parameters were: laser power, 60%, voltage, 25 kV, and mass difference in linear mode with external calibration, <6100 ppm (<60.01%). The matrix was sinapinic acid. The external calibration standard consisted of insulin, ubiquitin, cytochrome C, and myoglobin. Edman degradation was performed by Intas Pharma (http://intaspharma.com/).

## Supporting Information

Figure S1 **Linear regression for the inhibition of PI-PLC activity.** (a) inhibition of PI-PLC activity on phosphatidylinositol (PI) by trypsin inhibitor AEBSF. (b) inhibition of PI-PLC activity on PI and phosphatidylcholine (PC), cholesterol (CH), and phosphatidylethanolamine (PE) by trypsin inhibitor AEBSF.
(PDF)

Table S1 **Percentage identity/similarity among all proteases chosen for the search module and the PI and PC PLC from** ***Bacillus cereus*** **.**
(PDF)

## References

[1] Walsh KA, Neurath H (1964) Trypsinogen and chymotrypsinogen as homologous proteins. Proc Natl Acad Sci USA 52: 884–889. 14224394 10.1073/pnas.52.4.884 PMC300366

[2] Rawlings ND, Barrett AJ (1993) Evolutionary families of peptidases. Biochem J 290 (Pt 1): 205–218. 10.1042/bj2900205 PMC1132403 8439290

[3] Rawlings ND, Barrett AJ, Bateman A (2012) MEROPS: the database of proteolytic enzymes, their substrates and inhibitors. Nucleic Acids Res 40: D343–350. 22086950 10.1093/nar/gkr987 PMC3245014

[4] Lopez-Otin C, Bond JS (2008) Proteases: multifunctional enzymes in life and disease. J Biol Chem 283: 30433–30437. 18650443 10.1074/jbc.R800035200 PMC2576539

[5] Chakraborty S, Minda R, Salaye L, Bhattacharjee SK, Rao BJ (2011) Active site detection by spatial conformity and electrostatic analysis-unravelling a proteolytic function in shrimp alkaline phosphatase. PLoS ONE 6: e28470. 22174814 10.1371/journal.pone.0028470 PMC3234256

[6] Chakraborty S, Rao BJ (2012) A measure of the promiscuity of proteins and characteristics of residues in the vicinity of the catalytic site that regulate promiscuity. PLoS ONE 7: e32011. 22359655 10.1371/journal.pone.0032011 PMC3281107

[7] Rawlings ND, Morton FR (2008) The MEROPS batch BLAST: a tool to detect peptidases and their non-peptidase homologues in a genome. Biochimie 90: 243–259. 17980477 10.1016/j.biochi.2007.09.014

[8] Altschul SF, Madden TL, Schaffer AA, Zhang J, Zhang Z, et al (1997) Gapped BLAST and PSI- BLAST: a new generation of protein database search programs. Nucleic Acids Res 25: 3389–3402. 9254694 10.1093/nar/25.17.3389 PMC146917

[9] Russell RB (1998) Detection of protein three-dimensional side-chain patterns: new examples of convergent evolution. J Mol Biol 279: 1211–1227. 9642096 10.1006/jmbi.1998.1844

[10] Kraut J (1977) Serine proteases: structure and mechanism of catalysis. Annu Rev Biochem 46: 331–358. 332063 10.1146/annurev.bi.46.070177.001555

[11] Doolittle RF (1994) Convergent evolution: the need to be explicit. Trends Biochem Sci 19: 15–18. 8140615 10.1016/0968-0004(94)90167-8

[12] Gherardini PF, Wass MN, Helmer-Citterich M, Sternberg MJ (2007) Convergent evolution of enzyme active sites is not a rare phenomenon. J Mol Biol 372: 817–845. 17681532 10.1016/j.jmb.2007.06.017

[13] Holm L, Kaariainen S, Rosenstrom P, Schenkel A (2008) Searching protein structure databases with DaliLite v.3. Bioinformatics 24: 2780–2781. 18818215 10.1093/bioinformatics/btn507 PMC2639270

[14] Chou KC, Cai YD (2006) Prediction of protease types in a hybridization space. Biochem Biophys Res Commun 339: 1015–1020. 16325146 10.1016/j.bbrc.2005.10.196

[15] Shen HB, Chou KC (2009) Identification of proteases and their types. Anal Biochem 385: 153–160. 19007742 10.1016/j.ab.2008.10.020

[16] van Loo B, Jonas S, Babtie AC, Benjdia A, Berteau O, et al (2010) An efficient, multiply promiscuous hydrolase in the alkaline phosphatase superfamily. Proc Natl Acad Sci USA 107: 2740–2745. 20133613 10.1073/pnas.0903951107 PMC2840280

[17] Brien PJ, Herschlag D (1999) Catalytic promiscuity and the evolution of new enzymatic activities. Chem Biol 6: R91–R105. 10099128 10.1016/S1074-5521(99)80033-7

[18] Helland R, Larsen RL, Asgeirsson B (2009) The 1.4 Å crystal structure of the large and cold-active *Vibrio sp*. alkaline phosphatase. Biochim Biophys Acta 1794: 297–308. 18977465 10.1016/j.bbapap.2008.09.020

[19] Chakraborty S, Asgeirsson B, Minda R, Salaye L, Frere JM, et al (2012) Inhibition of a cold-active alkaline phosphatase by imipenem revealed by *in silico* modeling of metallo-β-lactamase active sites. FEBS Lett 586: 3710–3715. 22982109 10.1016/j.febslet.2012.08.030

[20] Ingmer H, Brondsted L (2009) Proteases in bacterial pathogenesis. Res Microbiol 160: 704–710. 19778606 10.1016/j.resmic.2009.08.017

[21] Kadurugamuwa JL, Beveridge TJ (1995) Virulence factors are released from *Pseudomonas aeruginosa* in association with membrane vesicles during normal growth and exposure to gentamicin: a novel mechanism of enzyme secretion. J Bacteriol 177: 3998–4008. 7608073 10.1128/jb.177.14.3998-4008.1995 PMC177130

[22] Songer JG (1997) Bacterial phospholipases and their role in virulence. Trends Microbiol 5: 156–161. 9141190 10.1016/S0966-842X(97)01005-6

[23] Katan M (1998) Families of phosphoinositide-specific phospholipase C: structure and function. Biochim Biophys Acta 1436: 5–17. 9838022 10.1016/s0005-2760(98)00125-8

[24] Goni FM, Montes LR, Alonso A (2012) Phospholipases C and sphingomyelinases: Lipids as substrates and modulators of enzyme activity. Prog Lipid Res 51: 238–266. 22504350 10.1016/j.plipres.2012.03.002

[25] Camilli A, Goldfine H, Portnoy DA (1991) *Listeria* monocytogenes mutants lacking phosphatidylinositol-specific phospholipase C are avirulent. J Exp Med 173: 751–754. 1847723 10.1084/jem.173.3.751 PMC2118838

[26] Griffith OH, Ryan M (1999) Bacterial phosphatidylinositol-specific phospholipase C: structure, func- tion, and interaction with lipids. Biochim Biophys Acta 1441: 237–254. 10570252 10.1016/s1388-1981(99)00153-5

[27] Pomerantsev AP, Kalnin KV, Osorio M, Leppla SH (2003) Phosphatidylcholine-specific phospholipase C and sphingomyelinase activities in bacteria of the *Bacillus cereus* group. Infect Immun 71: 6591–6606. 14573681 10.1128/IAI.71.11.6591-6606.2003 PMC219565

[28] Rosenblum JS, Kozarich JW (2003) Prolyl peptidases: a serine protease subfamily with high potential for drug discovery. Curr Opin Chem Biol 7: 496–504. 12941425 10.1016/s1367-5931(03)00084-x

[29] Schroder HK, Willassen NP, Smalas AO (1998) Structure of a non-psychrophilic trypsin from a cold- adapted fish species. Acta Crystallogr D Biol Crystallogr 54: 780–798. 9757092 10.1107/s0907444997018611

[30] Heinz DW, Ryan M, Bullock TL, Griffith OH (1995) Crystal structure of the phosphatidylinositol- specific phospholipase C from *Bacillus cereus* in complex with myo-inositol. EMBO J 14: 3855–3863. 7664726 10.1002/j.1460-2075.1995.tb00057.x PMC394464

[31] Rout AK, Minda R, Peri D, Ramakrishnan V, Bhattacharjee SK, et al (2010) Sequence specific 1H, 13C and 15N backbone resonance assignments of UVI31+ from *Chlamydomonas reinhardtii* . Biomol NMR Assign 4: 171–174. 20526700 10.1007/s12104-010-9239-4

[32] Shukla M, Minda R, Singh H, Tirumani S, Chary KV, et al (2012) UVI31+ is a DNA endonuclease that dynamically localizes to chloroplast pyrenoids in *C. reinhardtii* . PLoS ONE 7: e51913. 23284814 10.1371/journal.pone.0051913 PMC3524116

[33] Titball RW, Leslie DL, Harvey S, Kelly D (1991) Hemolytic and sphingomyelinase activities of *Clostridium perfringens* alpha-toxin are dependent on a domain homologous to that of an enzyme from the human arachidonic acid pathway. Infect Immun 59: 1872–1874. 1902199 10.1128/iai.59.5.1872-1874.1991 PMC257931

[34] Jeffery CJ (2009) Moonlighting proteins–an update. Mol Biosyst 5: 345–350. 19396370 10.1039/b900658n

[35] Lodola A, Branduardi D, De Vivo M, Capoferri L, Mor M, et al (2012) A catalytic mechanism for cysteine N-terminal nucleophile hydrolases, as revealed by free energy simulations. PLoS ONE 7: e32397. 22389698 10.1371/journal.pone.0032397 PMC3289653

[36] Ekici OD, Paetzel M, Dalbey RE (2008) Unconventional serine proteases: variations on the catalytic Ser/His/Asp triad configuration. Protein Sci 17: 2023–2037. 18824507 10.1110/ps.035436.108 PMC2590910

[37] Rosenblum G, Van den Steen PE, Cohen SR, Bitler A, Brand DD, et al (2010) Direct visualization of protease action on collagen triple helical structure. PLoS ONE 5: e11043. 20585385 10.1371/journal.pone.0011043 PMC2886829

[38] Rawlings ND, Barrett AJ, Bateman A (2011) Asparagine peptide lyases: a seventh catalytic type of proteolytic enzymes. J Biol Chem 286: 38321–38328. 21832066 10.1074/jbc.M111.260026 PMC3207474

[39] Tajima N, Kawai F, Park SY, Tame JR (2010) A novel intein-like autoproteolytic mechanism in autotransporter proteins. J Mol Biol 402: 645–656. 20615416 10.1016/j.jmb.2010.06.068

[40] Song J, Tan H, Boyd SE, Shen H, Mahmood K, et al (2011) Bioinformatic approaches for predicting substrates of proteases. J Bioinform Comput Biol 9: 149–178. 21328711 10.1142/s0219720011005288

[41] Koblinski JE, Ahram M, Sloane BF (2000) Unraveling the role of proteases in cancer. Clin Chim Acta 291: 113–135. 10675719 10.1016/s0009-8981(99)00224-7

[42] Korkmaz B, Moreau T, Gauthier F (2008) Neutrophil elastase, proteinase 3 and cathepsin G: physic- ochemical properties, activity and physiopathological functions. Biochimie 90: 227–242. 18021746 10.1016/j.biochi.2007.10.009

[43] Lutgens SP, Cleutjens KB, Daemen MJ, Heeneman S (2007) Cathepsin cysteine proteases in cardio- vascular disease. FASEB J 21: 3029–3041. 17522380 10.1096/fj.06-7924com

[44] Quesada V, Ordonez GR, Sanchez LM, Puente XS, Lopez-Otin C (2009) The Degradome database: mammalian proteases and diseases of proteolysis. Nucleic Acids Res 37: D239–243. 18776217 10.1093/nar/gkn570 PMC2686449

[45] Shindo T, Misas-Villamil JC, Horger AC, Song J, van der Hoorn RA (2012) A role in immunity for *Arabidopsis* cysteine protease RD21, the ortholog of the tomato immune protease C14. PLoS ONE 7: e29317. 22238602 10.1371/journal.pone.0029317 PMC3253073

[46] Kunkel M, Vuyisich M, Gnanakaran G, Bruening GE, Dandekar AM, et al (2007) Rapid clearance of bacteria and their toxins: development of therapeutic proteins. Crit Rev Immunol 27: 233–245. 18197819 10.1615/critrevimmunol.v27.i3.40

[47] Jackson MW, Silva-Herzog E, Plano GV (2004) The ATP-dependent ClpXP and Lon proteases regulate expression of the *Yersinia pestis* type III secretion system via regulated proteolysis of YmoA, a small histone-like protein. Mol Microbiol 54: 1364–1378. 15554975 10.1111/j.1365-2958.2004.04353.x

[48] Takaya A, Tabuchi F, Tsuchiya H, Isogai E, Yamamoto T (2008) Negative regulation of quorum-sensing systems in *Pseudomonas aeruginosa* by ATP-dependent Lon protease. J Bacteriol 190: 4181–4188. 18408026 10.1128/JB.01873-07 PMC2446771

[49] Tomoyasu T, Ohkishi T, Ukyo Y, Tokumitsu A, Takaya A, et al (2002) The ClpXP ATP-dependent protease regulates flagellum synthesis in *Salmonella enterica* serovar typhimurium. J Bacteriol 184: 645–653. 11790733 10.1128/JB.184.3.645-653.2002 PMC139528

[50] Krojer T, Sawa J, Schafer E, Saibil HR, Ehrmann M, et al (2008) Structural basis for the regulated protease and chaperone function of DegP. Nature 453: 885–890. 18496527 10.1038/nature07004

[51] Kruger E, Witt E, Ohlmeier S, Hanschke R, Hecker M (2000) The clp proteases of Bacillus subtilis are directly involved in degradation of misfolded proteins. J Bacteriol 182: 3259–3265. 10809708 10.1128/jb.182.11.3259-3265.2000 PMC94515

[52] Bomberger JM, Maceachran DP, Coutermarsh BA, Ye S, O’Toole GA, et al (2009) Long-distance delivery of bacterial virulence factors by *Pseudomonas aeruginosa* outer membrane vesicles. PLoS Pathog 5: e1000382. 19360133 10.1371/journal.ppat.1000382 PMC2661024

[53] Furuta N, Takeuchi H, Amano A (2009) Entry of *Porphyromonas gingivalis* outer membrane vesicles into epithelial cells causes cellular functional impairment. Infect Immun 77: 4761–4770. 19737899 10.1128/IAI.00841-09 PMC2772519

[54] Amano A, Takeuchi H, Furuta N (2010) Outer membrane vesicles function as offensive weapons in host-parasite interactions. Microbes Infect 12: 791–798. 20685339 10.1016/j.micinf.2010.05.008

[55] Ellis TN, Kuehn MJ (2010) Virulence and immunomodulatory roles of bacterial outer membrane vesicles. Microbiol Mol Biol Rev 74: 81–94. 20197500 10.1128/MMBR.00031-09 PMC2832350

[56] Li Z, Clarke AJ, Beveridge TJ (1998) Gram-negative bacteria produce membrane vesicles which are capable of killing other bacteria. J Bacteriol 180: 5478–5483. 9765585 10.1128/jb.180.20.5478-5483.1998 PMC107602

[57] Chakraborty S (2012) Enumerating pathways of proton abstraction based on a spatial and electrostatic analysis of residues in the catalytic site. PLOS ONE 7: e39577. 22745790 10.1371/journal.pone.0039577 PMC3379984

[58] Chakraborty S (2013) A quantitative measure of electrostatic perturbation in holo and apo enzymes induced by structural changes. PLoS ONE 8: e59352. 23516628 10.1371/journal.pone.0059352 PMC3597595

[59] Chakraborty S (2012) An automated flow for directed evolution based on detection of promiscuous scaffolds using spatial and electrostatic properties of catalytic residues. PLOS ONE 7: e40408. 22811760 10.1371/journal.pone.0040408 PMC3394801

[60] Chakraborty S, Minda R, Salaye L, Dandekar AM, Bhattacharjee SK, et al (2013) Promiscuity-based enzyme selection for rational directed evolution experiments. Methods Mol Biol 978: 205–216. 23423899 10.1007/978-1-62703-293-3_15

[61] Chakraborty S., Rao B.J., Baker N., Ásgeirsson B. Structural phylogeny by profile extraction and multiple superimposition using electrostatic congruence as a discriminator. Intrinsically Disordered Proteins 1: e25463. 10.4161/idp.25463 PMC4212511 25364645

[62] Lone AM, Nolte WM, Tinoco AD, Saghatelian A (2010) Peptidomics of the prolyl peptidases. AAPS J 12: 483–491. 20552307 10.1208/s12248-010-9208-y PMC2976987

[63] Titball RW (1993) Bacterial phospholipases C. Microbiol Rev. 57: 347–366. 10.1128/mr.57.2.347-366.1993 PMC372913 8336671

[64] Page MJ, Di Cera E (2008) Evolution of peptidase diversity. J Biol Chem 283: 30010–30014. 18768474 10.1074/jbc.M804650200 PMC2573091

[65] Titball RW, Naylor CE, Basak AK (1999) The *Clostridium perfringens* alpha-toxin. Anaerobe 5: 51–64. 16887662 10.1006/anae.1999.0191

[66] McDonel JL (1980) *Clostridium perfringens* toxins (type A, B, C, D, E). Pharmacol Ther 10: 617–655. 6255496 10.1016/0163-7258(80)90031-5

[67] Sakurai J, Nagahama M, Oda M (2004) *Clostridium perfringens* alpha-toxin: characterization and mode of action. J Biochem 136: 569–574. 15632295 10.1093/jb/mvh161

[68] Naylor CE, Eaton JT, Howells A, Justin N, Moss DS, et al (1998) Structure of the key toxin in gas gangrene. Nat Struct Biol 5: 738–746. 9699639 10.1038/1447

[69] Guillouard I, Alzari PM, Saliou B, Cole ST (1997) The carboxy-terminal C2-like domain of the alpha- toxin from *Clostridium perfringens* mediates calcium-dependent membrane recognition. Mol Microbiol 26: 867–876. 9426125 10.1046/j.1365-2958.1997.6161993.x

[70] Nagahama M, Mukai M, Morimitsu S, Ochi S, Sakurai J (2002) Role of the C-domain in the biological activities of *Clostridium perfringens* alpha-toxin. Microbiol Immunol 46: 647–655. 12477243 10.1111/j.1348-0421.2002.tb02748.x

[71] Lobkovsky E, Moews PC, Liu H, Zhao H, Frere JM, et al (1993) Evolution of an enzyme activity: crystallographic structure at 2 Å resolution of cephalosporinase from the ampC gene of *Enterobacter cloacae* P99 and comparison with a class A penicillinase. Proc Natl Acad Sci USA 90: 11257–11261. 8248237 10.1073/pnas.90.23.11257 PMC47961

[72] Gosalia DN, Salisbury CM, Ellman JA, Diamond SL (2005) High throughput substrate specificity profiling of serine and cysteine proteases using solution-phase fluorogenic peptide microarrays. Mol Cell Proteomics 4: 626–636. 15705970 10.1074/mcp.M500004-MCP200

[73] Boulware KT, Daugherty PS (2006) Protease specificity determination by using cellular libraries of peptide substrates (CLiPS). Proc Natl Acad Sci USA 103: 7583–7588. 16672368 10.1073/pnas.0511108103 PMC1456804

[74] Zhao H, Arnold FH (1999) Directed evolution converts subtilisin E into a functional equivalent of thermitase. Protein Eng 12: 47–53. 10065710 10.1093/protein/12.1.47

[75] Cheng K, Lu F, Li M, Liang X (2010) Improvement of subtilisin-like serine alkaline protease by directed evolution for cold-adaptation. In: Bioinformatics and Biomedical Engineering (iCBBE), 2010 4th International Conference on. 1–4. doi:10.1109/ICBBE.2010.5517430.

[76] Venekei I, Szilagyi L, Graf L, Rutter WJ (1996) Attempts to convert chymotrypsin to trypsin. FEBS Lett 383: 143–147. 8612781

[77] Baker NA, Sept D, Joseph S, Holst MJ, McCammon JA (2001) Electrostatics of nanosystems: appli- cation to microtubules and the ribosome. Proc Natl Acad Sci USA 98: 10037–10041. 11517324 10.1073/pnas.181342398 PMC56910

[78] Dolinsky TJ, Nielsen JE, McCammon JA, Baker NA (2004) PDB2PQR: an automated pipeline for the setup of Poisson-Boltzmann electrostatics calculations. Nucleic Acids Res 32: W665–667. 15215472 10.1093/nar/gkh381 PMC441519

[79] Ahyayauch H, Villar AV, Alonso A, Goni FM (2005) Modulation of PI-specific phospholipase C by membrane curvature and molecular order. Biochemistry 44: 11592–11600. 16114896 10.1021/bi050715k

[80] Villar AV, Alonso A, Goni FM (2000) Leaky vesicle fusion induced by phosphatidylinositol-specific phospholipase C: observation of mixing of vesicular inner monolayers. Biochemistry 39: 14012–14018. 11087348 10.1021/bi992515c

