# Supplementary material for: A Computational Module Assembled from Different Protease Family Motifs Identifies PI PLC from Bacillus cereus as a Putative Prolyl Peptidase with a Serine Protease Scaffold
Source: PLoS One. 2013 Aug 5;8(8):e70923. doi: 10.1371/journal.pone.0070923 (PMC3733634; doi:10.1371/journal.pone.0070923)
Supplement: Figure S1 — Linear regression for the inhibition of PI-PLC activity. (a) inhibition of PI-PLC activity on phosphatidylinositol (PI) by trypsin inhibitor AEBSF. (b) inhibition of PI-PLC activity on PI and phosphatidylcholine (PC), cholesterol (CH), and phosphatidylethanolamine (PE) by trypsin inhibitor AEBSF. (PDF) [file pone.0070923.s001.pdf]

CONDITIONS	AEBSF (mM)	SLOPE	b	R^2
PI + PLC	0	0.030	-0.118	0.996
PI + PLC	1.0	0.024	-0.147	0.999

(a)

CONDITIONS	AEBSF (mM)	SLOPE	b	R^2
PI/PE/PC/CH + PLC	0	0.322	-0.060	0.993
PI/PE/PC/CH + PLC	0.05	0.032	-0.069	0.996
PI/PE/PC/CH + PLC	0.1	0.022	-0.016	0.977

(b)

Supplementary Fig. 1: **Linear regression for the inhibition of PI-PLC activity : (a)** The inhibition of PI-PLC activity on phosphatidylinositol (PI) by trypsin inhibitor AEBSF. **(b)** The inhibition of PI-PLC activity on PI in addition to phosphatidylcholine (PC), cholesterol (CH) and phosphatidylethanolamine (PE) by trypsin inhibitor AEBSF.
